# Herbal compound “Songyou Yin” attenuates hepatoma cell invasiveness and metastasis through downregulation of cytokines secreted by activated hepatic stellate cells

**DOI:** 10.1186/1472-6882-13-89

**Published:** 2013-04-27

**Authors:** Qing-An Jia, Zhi-Ming Wang, Zheng-Gang Ren, Yang Bu, Xiao-Ying Xie, Yan-Hong Wang, Lan Zhang, Qiang-Bo Zhang, Tong-Chun Xue, Li-Fen Deng, Zhao-You Tang

**Affiliations:** 1 Liver Cancer Institute, Zhongshan Hospital, Fudan University; Key Laboratory of Carcinogenesis and Cancer Invasion, Ministry of Education, Shanghai 200032, China; 2 Institutes of Biomedical Sciences, Fudan University, Shanghai 200032, China

**Keywords:** Hepatocellular carcinoma, Herbal compound, Hepatic stellate cells, Metastasis

## Abstract

**Background:**

Activated hepatic stellate cells (aHSCs) play an important role in the progression of hepatocellular carcinoma (HCC). Here, we determined if cytokines secreted in response to the herbal compound “Songyou Yin” (SYY) treatment of aHSCs could influence invasiveness and metastatic capabilities of hepatoma cells.

**Methods:**

Primary rat hepatic stellate cells (HSCs) were isolated, activated, divided into SYY treated and untreated (nSYY) groups, and conditioned media (CM-SYY and CM-nSYY, respectively) were collected. The hepatoma cell line, McA-RH7777 was cultured for 4 weeks with SYY, CM-SYY, and CM-nSYY, designated McA-SYY, McA-SYYCM and McA-nSYYCM. The invasiveness and metastatic capabilities were evaluated using Matrigel invasion assay *in vitro* and pulmonary metastasis *in vivo*. Matrix metalloproteinase-2 (MMP-2), MMP-9, E-cadherin, N-cadherin, and vimentin protein levels in McA-SYYCM and McA-nSYYCM were evaluated by Western blot. Cytokine levels in conditioned media were tested using enzyme-linked immunosorbent assay (ELISA).

**Results:**

Matrigel invasion assay indicated that the number of McA-SYYCM cells passing through the basement membrane was less than in McA-nSYYCM cells (*P* < 0.01). Similar results were also observed *in vivo* for lung metastasis. McA-SYYCM cells showed less pulmonary metastasis capabilities than McA-nSYYCM cells (*P* < 0.001). The reduced expression of MMP-2 and reversed epithelial to mesenchymal transition with E-cadherin upregulation, and N-cadherin and vimentin downregulation were also found in McA-SYYCM compared to McA-nSYYCM. Metastasis-promoting cytokines hepatocyte growth factor, interleukin-6, transforming growth factor-β1, and vascular endothelial growth factor were markedly decreased in CM-SYY compared to CM-nSYY.

**Conclusions:**

SYY attenuates hepatoma cell invasiveness and metastasis capabilities through downregulating cytokines secreted by activated hepatic stellate cells.

## Background

Liver cancer (mainly hepatocellular carcinoma, HCC) in men is the fifth most frequently diagnosed cancer worldwide, but the second most frequent cause of cancer death [1]. The majority of HCC patients have a background of chronic liver disease that primarily develops in cirrhosis, such as chronic infection by hepatitis B virus and hepatitis C virus, alcoholic injury [2]. Moreover, HCC may complicate non-alcoholic fatty liver disease (NAFLD) with mild or absent fibrosis, greatly expanding the population of HCC patients potentially at higher risk [3]. Activated hepatic stellate cells (aHSCs) have a key role in fibrogenesis and in filtrates of the HCC stroma, where they could play a critical role in HCC progression. Therefore, aHSCs are recognized as central in the development and progression of hepatocellular carcinoma [4,5]. Unfortunately, only limited therapeutic options are currently available for this condition.

Oriental herbal medicines have been used to treat malignancies since ancient times [6]. Experimental studies showed that extracts from herbal medicines had anticancer potential [7-13]. Bu-Zhong-Yi-Qi Tang, a mixture of ten herbs was found to suppress the growth of hepatoma cells [14]. Sho-Saiko-To, a compound of seven herbs was reported to inhibit proliferation of KIM-1 human hepatoma cells [15]. Recent studies revealed that the anticancer activity of herbal medicines was linked to modulation of apoptotic signaling, the cell cycle, angiogenic factors, invasion, and metastatic events in cancer cells [16-19]. We have recently reported that Songyou Yin (SYY), a mixture of five herbs inhibited HCC growth, prolonged survival by inducing tumor apoptosis associated with caspase-3 activation, and inhibited invasiveness associated with downregulation of matrix metalloproteinase-2 (MMP-2) [20]. Xiong et al. [21] found that increased HCC metastatic potential after oxaliplatin treatment could be attenuated by SYY. Although the tumor suppressive effects of SYY on HCC have been confirmed, the relationship between SYY and aHSCs, which are the most important mesenchymal cells in the tumor microenvironment, has not yet been established.

Hepatic stellate cells (HSCs) are activated in response to liver damage, and can transdifferentiate into myofibroblasts (MFs), leading to the development of hepatic fibrosis [22,23]. Recent literature has highlighted the cross-talk between tumor cells and aHSCs in the pathogenesis of HCC. Wang et al. [24] demonstrated that cancer associated fibroblasts (CAFs) obtained from lung cancer tissue produced hepatocyte growth factor (HGF) which activated the c-Met pathway, leading to invasion and metastasis in cancer cells. Studebaker et al. [25] reported that fibroblasts isolated from breast cancer could enhance cancer cell invasiveness in an interleukin-6 (IL-6) dependent manner. Mazzocca et al. [26] found that transforming growth factor-β (TGF-β) receptor inhibitor LY2109761 could inhibit production of TGF-β secreted by CAFs, and block the cross-talk between CAFs and HCC, and thus inhibit the progression of HCC. Therefore, prevention of aHSCs in the tumor microenvironment may be a potential strategy to prevent and treat HCC.

In the present study, we determined if SYY could affect growth factors secreted by aHSCs, block the cross-talk between aHSCs and HCC, and indirectly influence the invasiveness and metastatic potential of hepatoma cells.

## Methods

### Characterization and preparation of herbal extracts

The Chinese herbal medicine formula SYY is a dietary component authorized by the Chinese State Food and Drug Administration (Grant No. G20070160), which includes five Chinese medicinal herbal extracts with fingerprint in the following proportions (w/w): *Salvia miltiorrhiza* Bge., 14.3%; *Astragalus membranaceus* Bge., 14.3%; *Lycium barbarum* L., 23.8%; *Crataegus pinnatifida* Bge., 23.8% and *Trionyx sinensis* Wiegmann, 23.8% (all from China) [20]. SYY used *in vitro* with the same batch number (#20110401) was produced by Shanghai Fang Xin Pharmaceutical Technology Co., Ltd. (Shanghai, China). The 800 mg/ml SYY preparation was sterilized with two 0.22 μm filtrations (Millipore, Billerica, MA, USA) and prepared for further use *in vitro.*

### Animals

Twelve-week-old inbred male Buffalo rats (300 g ± 20 g) used in this study were obtained from the Chinese Academy of Science and maintained under pathogen free conditions. The experimental protocol was approved by the Shanghai Medical Experimental Animal Care Commission.

### Isolation and preparation of rat liver HSCs

Rat liver HSCs were isolated by collagenase digestion and purified by density gradient centrifugation using Percoll (Sigma-Aldrich, St. Louis, MO, USA), as previously described [27] with slight modifications. The liver was perfused through the portal vein with buffer I [D-Hanks (Invitrogen, Grand Island, NY, USA) media containing 0.05% collagenase IV (Sigma-Aldrich), 0.1% pronase E (Roche, Madison, WI, USA)] at 37°C for 20 min. The liver was then excised and cut into small pieces in collagenase buffer containing 0.05% collagenase IV, 0.02% pronase E, 0.01% DNase (Sigma-Aldrich) for an additional 30 min. The suspension was filtered through nylon gauze and the filtrate was sedimented twice by centrifugation at 70 × *g* for 5 min at 4°C to remove parenchymal cells. The HSCs fraction in the supernatant was washed with D-Hanks buffer and collected by centrifugation at 650 × *g* for 5 min at 4°C. Cells were resuspended in DMEM (Invitrogen) and purified by flotation on a density cushion of Percoll (30%) by centrifugation at 1800 × *g* for 20 min at 4°C. The HSCs fraction was collected, sedimented at 650 × *g* for 7 min, and then resuspended in DMEM containing 10% fetal bovine serum (FBS).

### Preparation of conditioned media (CM)

Freshly isolated HSCs were cultured in high glucose DMEM containing 10% (v/v) FBS. Two weeks later, HSCs were activated and transferred into T75 flasks (1 × 10^6^ cells). The next day, the cultures were removed and 12 ml fresh serum free DMEM medium was added to the SYY sample (containing 2 mg/ml SYY) and control sample (no SYY). Twenty-four hours later, the cultures were centrifuged at 1000 × *g* and the supernatants were collected and designated CM-SYY (with SYY) and CM-nSYY (no SYY). All culture reagents were purchased from Invitrogen.

### Lactate dehydrogenase (LDH) cytotoxicity assay

The cytotoxicity detection kit ^PLUS^ (Roche) is a precise and fast colorimetric assay for quantitating cytotoxicity by measuring lactic dehydrogenase (LDH) activity released from damaged cells. The aHSCs were cultured and digested when the density reached 80%. After trypsin digestion, the cells were counted and pipetted into 96-well plates at 1000 cells/well. On the same plate, background controls (medium only); low controls (spontaneous LDH release), high controls (maximum LDH release), and experimental samples (2 mg/ml SYY) were prepared according to the manufacturer’s instructions. The 96-well plates were incubated in a humidified incubator at 37°C in 5% CO_2_ for 4 h, 8 h, and 12 h. Results were expressed as the mean absorbance of wells in groups at 492 nm. Cytotoxicity (%) was calculated using the equation: (experimental value − low control) / (high control − low control) × 100%.

### Immunofluorescence and western blot assays

The expression of α-smooth muscle actin (α-SMA) (Abcam, Cambridge, MA, USA) and desmin (Epitomics, Burlingame, CA, USA) were determined by immunofluorescence as previously described [28]. The aHSCs were grown on glass cover slips to 20 − 30% confluency, and then fixed, permeabilized, blocked, and incubated with α-SMA or desmin overnight at 4°C. Slides were then washed and incubated with FITC-conjugated secondary antibody (Jackson Labs, Bar Harbor, ME, USA). Cells were counterstained with 4'-6-diamidino-2-phenylindole (DAPI) to visualize cell nuclei and detected by fluorescence microscopy (Olympus, Tokyo, Japan). The protein expression of MMP-2, MMP-9, E-cadherin, N-cadherin, vimentin, and actin in McA-SYYCM and McA-nSYYCM conditioned media were detected by western blot assays as previously described with slight modifications [28]. And the primary antibodies were diluted according to the manufacturer’s instructions (Epitomics). The concentration of protein extracted from the McA-SYYCM and McA-nSYYCM were determined by the BCA Protein Assay Kit (Beyotime, Shanghai, China).

### Cells and cell cultures

The hepatoma cell line McA-RH7777 originally derived from the Buffalo rat was obtained from the American Type Culture Collection (Manassas, VA, USA). To determine the effect of conditioned media (CM) from SYY treated and untreated aHSCs, McA-RH7777 cells were cultured and passaged (every 4–5 days) in CM-nSYY (added with 10% FBS) and CM-SYY (added with 10% FBS and 2 mg/ml SYY) for 4 weeks and designated McA-SYYCM and McA-nSYYCM. McA-RH7777 cells cultured and passaged (every 4–5 days) in DMEM containing 10% FBS and 2 mg/ml SYY for 4 weeks were named McA-SYY (no CM added).

### Cell invasion assays

Cell invasion was assessed by transwell assays as described previously [29] (Boyden chambers, Corning, Flintshire, UK) using McA-SYY, McA-SYYCM, and McA-nSYYCM conditioned media. Eighty μl of Matrigel (1:8 dilution with DMEM) (BD Biosciences, San Jose, CA, USA) was added to each well 4 h before cells were seeded onto the membrane. Then, 5 × 10^4^ cells in serum free DMEM were seeded into the upper chamber of each well on the membrane (8.0 μm pore size) of a 24-well plate. DMEM containing 10% FBS was added to the lower chamber of each well. After 48 h, cells reaching the underside of the membrane were stained with Giemsa (Sigma-Aldrich) and counted at × 200 magnification.

### Enzyme-linked immunosorbent assay

Cytokines secreted into the media of CM-SYY and CM-nSYY were quantified by ELISA kits (R&D Labs, Minneapolis, MN, USA). Cytokines measured included HGF, IL-2, IL-6, IL-10, IL-12, matrix metalloproteinase-2 (MMP-2), MMP-9, transforming growth factor-β (TGF-β), tumor necrosis factor-α (TNF-α), and vascular endothelial growth factor (VEGF). Assays were performed according to the manufacturer’s instructions and conducted in quadruplicate which were described previously [30].

### *In vivo* evaluation of tumor metastasis

Four weeks after co-cultivation, McA-SYY, McA-SYYCM, and McA-nSYYCM cells were harvested, washed, and resuspended in PBS at 5 × 10^7^/ml. Then 5 × 10^6^ cells (0.1 ml cell suspension) were injected into the left lobe livers of 6-week-old Buffalo rats. Each group contained 6 rats. Four weeks later, animals were sacrificed and the numbers of lung metastatic nodules were evaluated by microscopy of serial sections of the right lung tissue block which is on behalf the entire pulmonary metastasis. And this method was reported by our group in previous research [31].

### Statistical analysis

Differences in the quantitative data obtained from cytokine expression levels, invasiveness, and pulmonary metastatic nodules among the three groups of hepatoma cells were evaluated by *t*-test. Statistical analysis was performed with SPSS 15.0 software for Windows (SPSS Inc. Chicago, IL, USA). *P* < 0.05 was considered as statistically significant.

## Results

### Isolation, activation, and identification of HSCs

**Figure 1:**
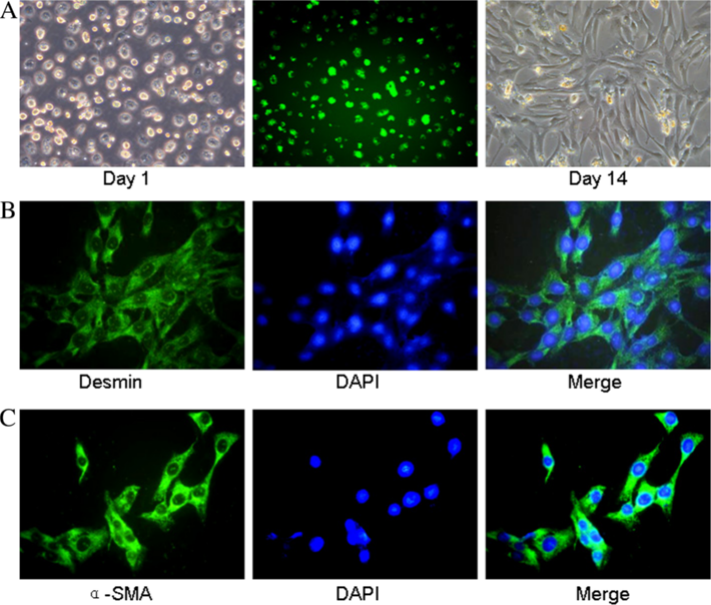
**Freshly isolated HSCs from buffalo rats were quiescent, rich in lipid droplets and with spontaneous green fluorescence.** Fourteen days after isolation, HSCs were activated and developed into fibroblast-like cells (**A**). Activated HSCs were immunostained with desmin (**B**), and α-SMA (**C**) followed by FITC-conjugated secondary antibody, and nuclei were stained with DAPI.

The results are presented in Figures 1A-1C. Hepatic stellate cells (HSCs) were isolated from Buffalo rat liver mesenchymal cells which were quiescent, rich in lipid droplets, and had spontaneous green fluorescence on the first day after isolation. Fourteen days later, HSCs developed into fibroblast-like cells (Figure 1A). The purity of aHSCs was approximately 95% as determined by desmin immunostaining, which only stains quiescent and activated HSCs (Figure 1B). The isolated HSCs were 100% activated with strong alpha smooth muscle actin (α-SMA) expression throughout the cells (Figure 1C).

### SYY exhibited no significant cytotoxicity in aHSC and HCC cell lines

**Figure 2:**
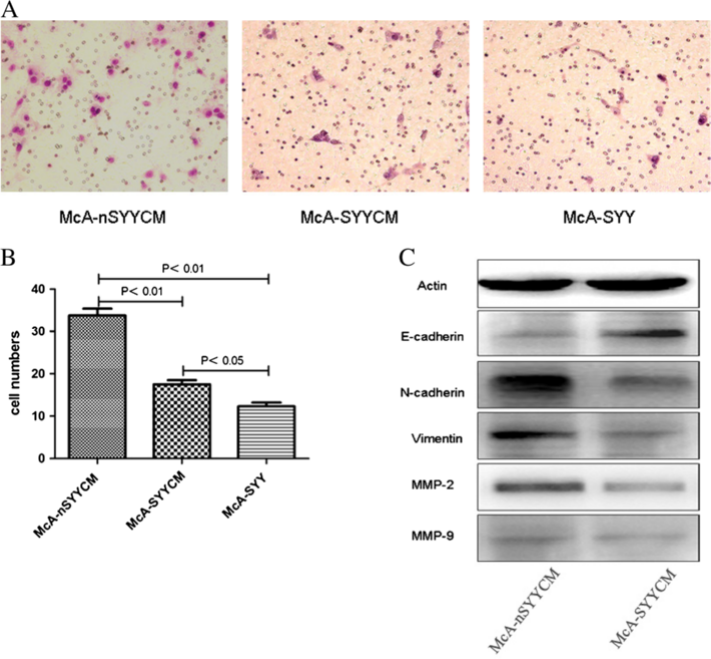
**The number of pulmonary nodules implanted with McA-SYYCM cells was less than the nodule number from McA-nSYYCM cells.** McA-SYY cell implants had the smallest number of nodules (**A**, **B**). The aHSCs and McA-RH7777 cells, which were treated with 2 mg/ml SYY for 4 h, 8 h, and 12 h, demonstrated no increased release of LDH, indicating no acute cytotoxicity (**C**).

The results are presented in Figure 2C. Activated Hepatic stellate cells (aHSCs) and the hepatoma cell line McA-RH7777 were treated with SYY 2 mg/ml for 0, 4, 8, 12 h demonstrated strange decline in release of lactate dehydrogenase (LDH) compared with the control group. When LDH release of the control group was set at 0%, the relative emission of LDH at 4, 8, 12 h was −7.07 ± 3.39%, -5.20 ± 2.17%, -5.76 ± 1.92% in aHSCs and −4.76 ± 1.32%, -6.04 ± 2.37%, -6.44 ± 1.97% in McA-RH7777. There was no statistically significant difference between the control and experimental groups (Figure 2C), indicating that SYY did not exhibit acute cytotoxicity.

### Hepatoma cells treated by conditioned media (CM) from SYY treated aHSCs showed reduced invasiveness

**Figure 3:**
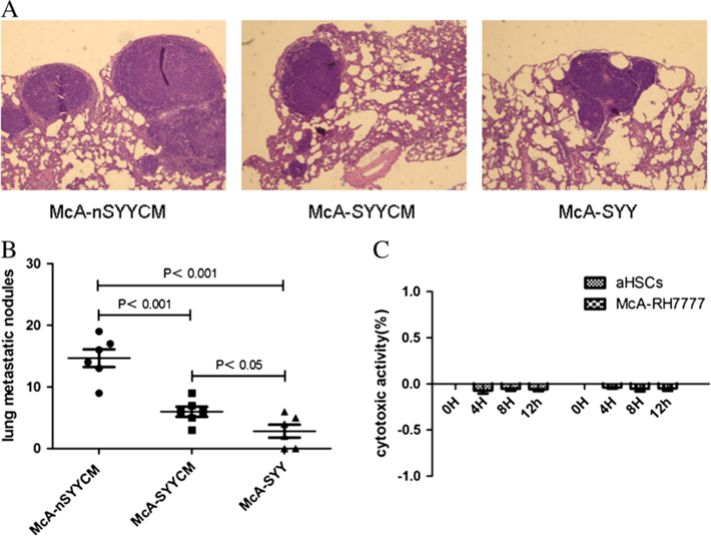
**The transwell assays demonstrated that McA-SYYCM cells passed through the basement membrane in less number than McA-nSYYCM cells, and McA-SYY cells were the lowest in number (A).** The number of invading cells is expressed as mean ± SD (**B**). Based upon western blots, E-cadherin was upregulated, while N-cadherin, vimentin, and MMP-2 were downregulated in McA-SYYCM cells compared to McA-nSYYCM cells. MMP-9 levels were low in both McA-SYYCM and McA-nSYYCM cells, and were not statistically different (**C**).

The results are presented in Figure 3A-3C. The cell invasion assay demonstrated that the number of McA-SYYCM cells passing through the basement membrane was less than the McA-nSYYCM cells (33.83 ± 3.87 *vs.* 17.50 ± 2.43, *P* < 0.01). There was only a small number of McA-SYY cells passing through the basement membrane, which was the lowest number of the three cell samples (12.33 ± 2.16, *P* < 0.05) (Figure 3A, 3B). Epithelial to mesenchymal transition (EMT) is characterized by the loss of the epithelial marker E-cadherin, and expression of mesenchymal markers such as N-cadherin and vimentin which is regarded as a critical characteristic of tumor invasion and metastasis. In the present study, E-cadherin upregulation with N-cadherin and vimentin downregulation were found in McA-SYYCM cells compared to McA-nSYYCM cells. MMP-2, as a predictor of recurrence and metastasis in cancer, was also found downregulated in McA-SYYCM cells. Expression of MMP-9 was low, with no significant differences in expression found in both McA-SYYCM and McA-nSYYCM cells (Figure 3C).

### Hepatoma cells treated by CM from SYY treated aHSCs showed reduced pulmonary metastasis

The results are presented in Figure 2A-2B. To investigate whether CM from SYY treated aHSCs could attenuate hepatoma cell pulmonary metastatic potential, three groups of cells (McA-SYY, McA-SYYCM, and McA-nSYYCM) were implanted into rat livers. Four weeks later, rats were sacrificed and the nodules of pulmonary metastasis were evaluated. The number of pulmonary nodules implanted with McA-SYYCM cells was reduced when compared to McA-nSYYCM cells (6.00 ± 2.00 *vs.* 14.67 ± 3.51, *P* < 0.001). In McA-SYY group there was only a small number of pulmonary nodules, which showed the lowest number of the three groups (2.83 ± 2.56, *P* < 0.05) (Figure 2A, 2B).

### SYY treatment altered the cytokines secretion of aHSCs

The results are presented in Table 1. Cytokines in CM collected from aHSCs could significantly induce proliferation and migration of hepatoma cells. Cytokines in CM from SYY treated aHSCs, which attenuate hepatoma cell invasiveness and metastasis, were measured by ELISA. In the conditioned media, HGF (144.62 ± 7.12 pg/ml *vs.* 115.49 ± 10.17 pg/ml, *P* = 0.0034), IL-6 (17.56 ± 0.33 pg/ml *vs.* 13.76 ± 1.41 pg/ml, *P* = 0.0215), TGF-β (20.42 ± 1.49 pg/ml *vs.* 17.69 ± 0.82 pg/ml, *P* = 0.0086), and VEGF (132.34 ± 8.27 pg/ml *vs.* 122.74 ± 10.62 ng/ml, *P* = 0.0481) were markedly decreased in CM-SYY compared to CM-nSYY (Table 1).

**Table 1:** Cytokine expression in CM-nSYY and CM-SYY using ELISA Assay

	***CM-nSYY *mean ± SD**	***CM-SYY *mean ± SD**	***P value***
HGF*	144.62 ± 7.12	115.49 ± 10.17	0.0034
IL-2*	14.28 ± 1.02	14.37 ± 0.42	0.860
IL-6*	17.56 ± 0.33	13.76 ± 1.41	0.0215
IL-10*	16.45 ± 1.88	17.56 ± 2.65	0.521
IL-12*	13.72 ± 0.69	13.50 ± 3.35	0.903
MMP-2**	23.10 ± 2.17	22.33 ± 1.63	0.590
TGF-β*	20.42 ± 1.49	17.69 ± 0.82	0.0086
TNF-a*	23.84 ± 3.24	23.01 ± 2.95	0.716
VEGF*	132.34 ± 8.27	122.74 ± 10.62	0.0481

^*^ pg/ml.

^**^ng/ml.

Data were obtained from 4 tests. Paired *t*-test was used for statistical analyses.

## Discussion

Liver fibrosis is strongly associated with HCC, with 90% of HCC cases arising in cirrhotic livers [32]. HSCs as one of the key cell types responsible for liver fibrosis were once known as lipocytes, Ito cells, or perisinusoidal cells. HSCs are activated in response to liver damage and transdifferentiate into MFs which lead to the development of hepatic fibrosis. They also infiltrate the stroma of liver tumors as CAFs promoting HCC cell proliferation and metastasis. Multiple mechanisms may be involved in the aHSCs which induce changes of the hepatic tumor phenotype. It is reported that aHSCs could interact with hepatoma cells via secretion of cytokines, extracellular matrix-mediated interactions, and direct cell-to-cell contacts [33]. In the present study, we found that SYY could indirectly attenuate hepatoma cell invasiveness and pulmonary metastasis through downregulation of cytokines secreted by aHSCs.

The development of distant metastasis requires the invasion of cancer cells from the primary tumor into the surrounding tissue. To acquire such invasive abilities, epithelial cancer cells must undergo several phenotypic changes, and aHSCs as one of the most important members in the tumor environment play a critical role in modulating the phenotypic changes. Amann et al. [34] demonstrated that CM collected from aHSCs could promote proliferation and migration of HCC cells. There are many cytokines secreted by aHSCs that are associated with tumor invasion and metastasis, ingcluding HGF, EGF, IL-1, IL-2, IL-6, MMP-2, MMP-9, TGF-β, TNF-α, VEGF and Wnt families [35-37]. Inhibition of TGF-β receptor activity interrupted the cross-talk between HCC cells and CAFs and therefore prevented tumor growth, intravasation and metastasis via inhibiting the proliferation of CAFs [26]. Studebaker et al. [25] found soluble IL-6 produced by issue-specific fibroblasts could promote growth and invasion of breast cancer cells which can be inhibited by the removal or inhibition of IL-6. Li et al. [38] found Wnt2 secreted by tumour fibroblasts promoted tumour progression in oesophageal cancer by activation of the Wnt/b-catenin signalling pathway. It was also reported that HGF upregulation promoted carcinogenesis and epithelial-mesenchymal transition in hepatocellular carcinoma via Akt and COX-2 pathways [39]. In the present study, we quantified ten cytokines secreted into the media of CM-SYY and CM-nSYY and found that HGF, IL-6, TGF-β, and VEGF were downregulated in CM from SYY treated aHSCs.

In the further study, we determined if CM from SYY treated aHSCs could block the cross-talk between aHSCs and HCC, and indirectly influence the invasiveness and metastatic potential of hepatoma cells. We cultured Buffalo rat hepatoma cells (McA-RH7777) in CM-SYY for four weeks and found reduced invasiveness and pulmonary metastasis capability compared to McA-nSYYCM cells (no SYY). EMT, which is regarded as a critical characteristic of tumor invasion and metastasis is characterized by the loss of epithelial marker E-cadherin and increased expression of mesenchymal markers such as N-cadherin and vimentin [40]. We found that EMT was reversed with the upregulation of E-cadherin and downregulation of N-cadherin and vimentin in McA-SYYCM compared to McA-nSYYCM cells. MMP-2 which is a predictor of recurrence and metastasis risk in cancer was also found downregulated in McA-SYYCM cells.

SYY is a Chinese herbal medicine formula consisting of five herbs. Some components of SYY such as tanshinone and astragalus saponins have demonstrated efficacy in treatment of malignancies [41,42]. We have shown that SYY could effectively inhibit tumor growth and metastasis, reverse the molecular changes consistent with EMT, and increase survival in a HCC nude mouse model [20,21]. In the present study, we found that SYY could attenuate hepatoma cell invasiveness and metastasis through downregulating cancer-promoting cytokines secreted by aHSCs. Recently, fibrosis-dependent tumorigenic mechanisms have been reported by many researchers [43,44]. However, studies which support anti-fibrotic therapeutic strategies to prevent and treat HCC are few. Treatment of the underlying cause of chronic liver injury and liver transplantation are the only two currently available therapeutic interventions capable of modifying liver fibrosis [45]. Elimination of the causative agent is often not possible, and liver transplantation has many drawbacks, including shortage of donors, costs, procedure-associated risks, and complications of immunosuppressive drugs. Our research suggests that traditional Chinese medicines, such as SYY may provide potential candidates to reduce liver cirrhosis and prevent liver tumors.

We have shown that SYY could attenuate hepatoma cell invasiveness and metastasis through downregulation of cytokines secreted by activated hepatic stellate cells. However, several fundamental questions remain to be answered concerning the ability of SYY to reverse liver fibrosis. Therefore, mouse models with a cirrhosis background should be established and the relationship among HCC, aHSCs, and SYY must be evaluated in future studies. Future studies will also determine if SYY can be useful in clinically treating human tumors.

## Conclusions

SYY attenuates hepatoma cell invasiveness and metastasis capabilities through downregulating cytokines secreted by activated hepatic stellate cells.

## Abbreviations

HCC: Hepatocellular carcinoma; HSCs: Hepatic stellate cells; SYY: Songyou Yin; CM: Conditioned media; ELISA: Enzyme-linked immunosorbent assay; aHSCs: Activated hepatic stellate cells; MFs: Myofibroblasts; CAFs: Cancer-associated fibroblasts; EMT: Epithelial to mesenchymal transition; TCM: Traditional Chinese medicine; HGF: Hepatocyte growth factor; IL-2: Interleukin-2; MMP-2: Matrix metalloproteinase-2; TGF-β: Transforming growth factor-β; TNF-α: Tumor necrosis factor-α; VEGF: Vascular endothelial growth factor.

## Competing interests

SYY formula was provided by professor Zhao-You Tang. SYY was produced and gifted by Shanghai Fang Xin Pharmaceutical Technology Co., Ltd. And Shanghai Fang Xin Pharmaceutical Technology Co., Ltd didn’t play any role in the funding, design, implementation or analysis of the study. The authors declare that they have no competing interests.

## Authors’ contributions

QAJ, ZMW, ZGR, YHW, YB, QBZ, LZ, YXX, TCX, LFD and ZYT contributed to the study design, analysis, and interpretation of data. ZYT and ZGR conceived the study. QAJ and ZMW performed the experiments. YB, LZ, YXX and QBZ participated in the isolation of HSCs. LFD participated in statistical analysis. QAJ drafted the manuscript. ZYT carried out the revision and provided important suggestions. All authors approved the final manuscript.

## Pre-publication history

The pre-publication history for this paper can be accessed here:

http://www.biomedcentral.com/1472-6882/13/89/prepub
